# Antineoplastic Agents Targeting Sphingolipid Pathways

**DOI:** 10.3389/fonc.2020.00833

**Published:** 2020-05-22

**Authors:** Alexander Kroll, Hwang Eui Cho, Min H. Kang

**Affiliations:** ^1^School of Medicine, Texas Tech University Health Sciences Center, Lubbock, TX, United States; ^2^Cancer Center, School of Medicine, Texas Tech University Health Sciences Center, Lubbock, TX, United States; ^3^Department of Pediatrics, School of Medicine, Texas Tech University Health Sciences Center, Lubbock, TX, United States

**Keywords:** sphingolipids, ceramides, sphingosine-1-phosphate, sphingomyelin, lipid biomarkers

## Abstract

Emerging studies in the enigmatic area of bioactive lipids have made many exciting new discoveries in recent years. Once thought to play a strictly structural role in cellular function, it has since been determined that sphingolipids and their metabolites perform a vast variety of cellular functions beyond what was previously believed. Of utmost importance is their role in cellular signaling, for it is now well understood that select sphingolipids serve as bioactive molecules that play critical roles in both cancer cell death and survival, as well as other cellular responses such as chronic inflammation, protection from intestinal pathogens, and intrinsic protection from intestinal contents, each of which are associated with oncogenesis. Importantly, it has been demonstrated time and time again that many different tumors display dysregulation of sphingolipid metabolism, and the exact profile of said dysregulation has been proven to be useful in determining not only the presence of a tumor, but also the susceptibility to various chemotherapeutic drugs, as well as the metastasizing characteristics of the malignancies. Since these discoveries surfaced it has become apparent that the understanding of sphingolipid metabolism and profile will likely become of great importance in the clinic for both chemotherapy and diagnostics of cancer. The goal of this paper is to provide a comprehensive review of the current state of chemotherapeutic agents that target sphingolipid metabolism that are undergoing clinical trials. Additionally, we will formulate questions involving the use of sphingolipid metabolism as chemotherapeutic targets in need of further research.

## Introduction to Sphingolipid Metabolism

**Figure 1:**
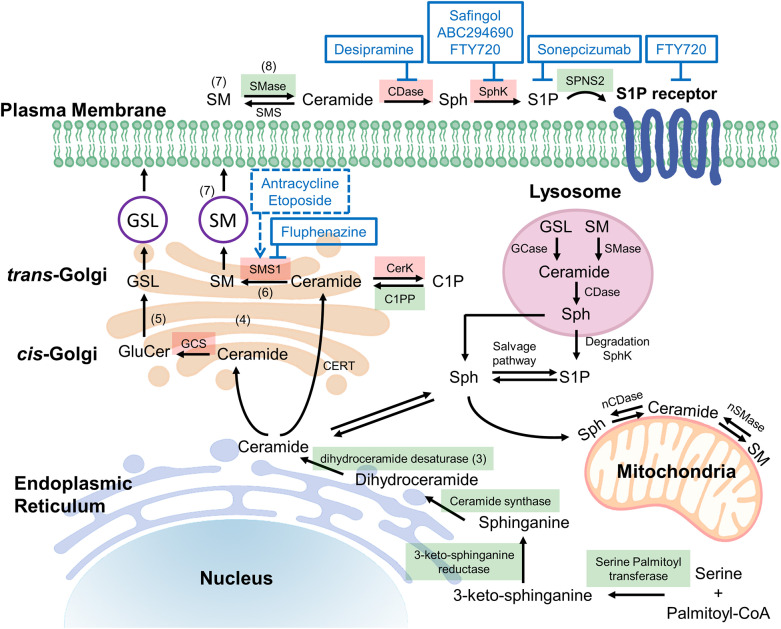
Potential targets in ceramide synthesis (sphingolipid) pathways. *De novo* ceramide synthesis begins at the endoplasmic reticulum (ER) with the condensation of serine and palmitoyl-CoA via serine palmitoyltransferase (SPT) forming 3-ketosphingosine, which is subsequently reduced by 3-ketoshinganine reductase (KSA reductase) to dihydrosphingosine. An acyl group is then linked via an amide bond by ceramide synthase (CerS 1-6) to form dihydroceramide, which is quickly dehydrated between carbons 4 and 5 by dihydroceramide desaturase (DES) to form ceramide (3). Once synthesized, ceramide may be translocated to the trans-golgi via ceramide transferase (CERT), at which it may be degraded, or reformed via salvage pathways (4). Alternatively, ceramide may diffuse to the cis-golgi at which it is converted into glucosylceramide (GluCer), a precursor for important fatty acids such as glycosphingolipids (GSL) and gangliosides (5). The action of sphingomyelin synthase 1 (SMS1) on ceramide at the trans-golgi results in the production of sphingomyelin (SM), composed of a long-chain sphingoid base, an amide-linked acyl chain and a phosphorylcholine headgroup (6). The isoenzymes differ in cellular location, SMS1 localized at the golgi whereas sphingomyelin synthase 2 (SMS2) may be found on the golgi or the plasma membrane (7). Acid sphingomyelinase (SMase) is an enzyme that converts sphingomyelin into ceramide, thus it is an important component of the rheostat. In response to apoptotic stimuli it is has been shown that phospholipid scrambling moves sequestered sphingomyelin from the outer leaflet to the cytosolic side of the plasma membrane such that sphingomyelinase may act on it, producing the apoptotic ceramide (8). The reverse of this process occurs via sphingomyelin synthase, thus to alter the rheostat to favor cell death, chemotherapeutic agents aim to induce sphingomyelinase and inhibit sphingomyelin synthase. Figure 1 has enzymes colored green and red to represent druggable targets that if inhibited, alter the rheostat to promote a pro-survival or pro-apoptotic cellular state respectively. C1P, ceramide-1-phosphate; C1PP, ceramide-1-phosphate phosphatase; CDase, ceramidase; CerK, ceramide kinase; GCase, glucocerebrosidase; GCS, glucosylceramide synthase; nCDase, neutral ceramidase; nSMase, neutral sphingomyelinase; S1P, Sphingosine-1-phosphate; Sph, sphingosine; SphK, sphingosine kinase.

**Figure 2:**
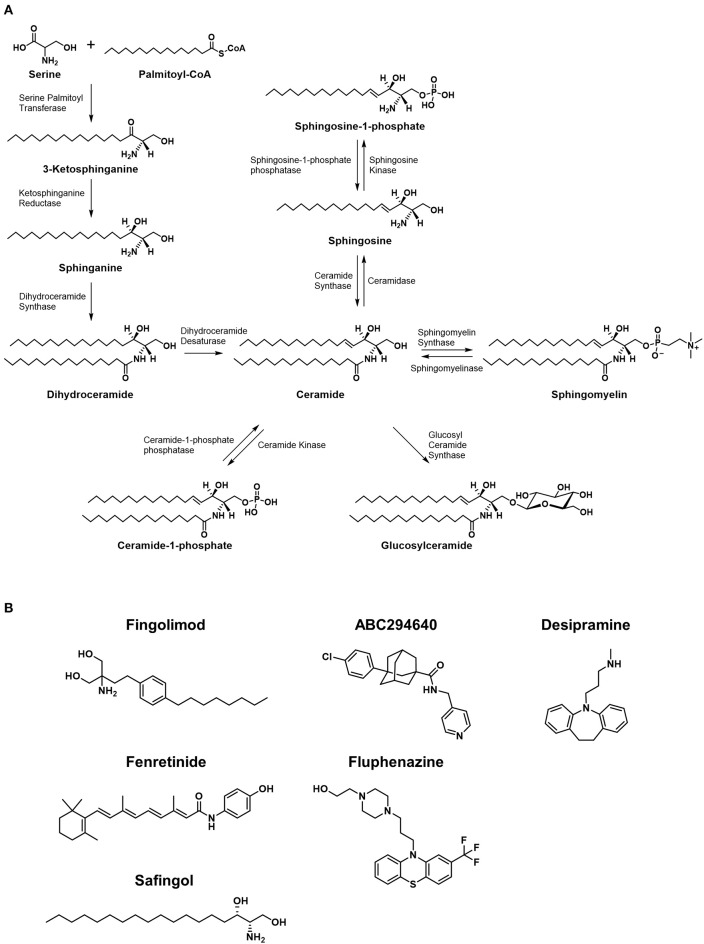
Metabolic pathways of sphingolipids and chemical structures of inhibitors of the pathways. **(A)** Major synthetic and metabolic pathways of sphingolipids. Increased ceramide leading to cytotoxicty comes from *de novo* synthesis resulted from stimulation of serine palmitoyltransferase and/or dihydroceramide synthase, or by degradation of sphingomyelins via spingomyelinases. The formation of ceramide-1-phosphate or glucosylceramide is considered shunting pathways to less toxic forms of sphingolipids. **(B)** The structures of small molecules that are currently under clinical investigation in cancer patients are shown.

Common to all sphingolipids are a sphingoid base and a fatty acid tail or head group attached to the base; the variations of each of these core components is what determines the ultimate function of each sphingolipid. For example, ceramides are a sphingolipid with the sphingoid base sphingosine together with a fatty acid tail, and they play an important function in the structure of cell membranes, but are also thought to be involved in cellular signaling regulating programmed cell death as well as cellular proliferation, making this type of sphingolipid an obvious target in treating cancer (1, 2). Ceramide lies at the intersection of all sphingolipid metabolism, for it is the addition of various headgroups that ultimately leads to more complex sphingolipids. Discussed in greater detail later in this review, a delicate balance between ceramide and its metabolites plays a crucial role in cellular fate, thus an understanding of the enzymes involved in the metabolism of sphingolipids is a critical concept to grasp when discussing targeting this pathway with anticancer agents. Figure 1

illustrates an abbreviated summary of some of the most relevant enzymes and sphingolipids involved in controlling the rheostat, therefore including many of the most promising chemotherapy targets (3–8). Figure 2A illustrates the molecular structures of many of the important lipids and metabolites being discussed.

### Bioactive—Ceramide, S1P Rheostat

**Figure 3:**
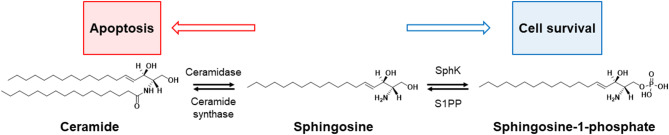
Rheostat of sphinglipid. The balance between cell survival and death (apoptosis) in sphingolipids is controlled by four enzymes: sphingosine kinase (SphK), sphingosine-1-phosphate phosphatase (S1PP), ceramidase, and ceramide synthase. The increase in ceramide turns up the rheostat toward apoptosis, and the increase in apoptotic precursors [e.g., sphingosine-1-phosphate (S1P)] toward cell survival.

Sphingosine-1-phosphate (S1P) and ceramide are bioactive lipids that are well known for their opposing roles on determining the fate of a cell. S1P plays a pro-survival role in cellular fate, while ceramide is known to be an apoptotic cellular messenger (3); the ratio of cellular levels between these two lipids is known as the sphingolipid rheostat, and this concept is illustrated in Figure 3.

While much of the focus in anticancer drug development targeting sphingolipid metabolism falls on the rheostat, the ratio of ceramide to S1P is not the only important balance of sphingolipids in cellular proliferation or death. As mentioned previously in the discussion of ceramide degradation and synthesis, metabolites such as glucosylceramide and ceramide-1-phosphate (C1P) are also critically balanced in cell fate, making the involved enzymes potential therapeutic targets, but these are only a few of many sphingolipid ratios that have been implicated in cancer pathogenesis. For example, studies have shown that the ratio of sphingosine to sphinganine may be involved in the pathogenesis of certain cancers, for this ratio has found to be altered in favor of sphinganine in rat nephromas (9) among others. Figure 2B shows the molecular structures of small molecules that are studied in clinical trials, which are discussed later in this review.

### Various Biomarkers in Cancer

A compelling verification in favor of the potential chemotherapeutic use of targeting sphingolipid metabolism are the numerous studies performed that have identified sphingolipid dysregulation in various malignancies. An interesting finding is that even though ceramide is directly converted to S1P, a small conversion in cellular ceramide exponentially increases the level of S1P, thus the rheostat is a critical, delicate balance controlling cellular fate (2). In prostate cancer patients, levels of both S1P and (sphingosine kinase 1) SphK1 have been identified as highly sensitive and specific biomarkers. Increased circulating plasma levels of S1P and increased activity of SphK1 correlated significantly with both the grade diagnosis of prostate cancer as well as the prognosis (10). Patients with hepatocellular carcinoma were shown to have increased SphK1/2 when compared with non-cancerous patients and increased SphK1 specifically was associated with a poorer prognosis (11). A similar finding was observed in hepatocellular carcinoma (HCC) patients' ceramide levels; ceramide levels in HCC tissue was lower than disease free tissue (12), but ceramide with a 16-carbon fatty acid chain (C16) and S1P was elevated in cancerous tissue when compared to cirrhotic liver tissue (13). Furthermore, C16 and C24 (with a 24-carbon fatty acid chain) ceramide levels were both shown to be elevated in pancreatic cancer patients with positive regional lymph node metastasis when compared to levels in patients with non-metastatic pancreatic cancer. Additionally, it was shown that sphingosine was elevated in pancreatic cancer patients compared to those without cancer, especially in more aggressive and advanced forms of cancer (14). Similar patterns of sphingolipid dysregulations used as biomarkers have been identified in HNSCC, gliomas, colon cancer breast cancer, and ovarian cancer; each of which implicates S1P, ceramides, SphK1, or the S1P receptor (S1PR) as an aberrant molecule that may be involved in the pathogenesis of each malignancy (15, 16).

### Exceptions to Rheostat

What has been presented thus far is an oversimplification of the rheostat involvement in tumorigenesis. For example, excessive ceramide accumulation has been found in both breast cancer (17) as well as highly invasive forms of pancreatic cancer (18). These contradictory findings may best be explained with a more detailed explanation of the varying enzymes responsible for generating ceramides with varying chain lengths. CERS1-6 are a set of six enzymes responsible for generating varying lengths of ceramide fatty acyl chains. Specifically, CERS1 and CERS 4 preferentially generate C18-20 ceramides, CERS5-6 generates C14-16 ceramides and CERS2 generates only C22-C24 ceramides. Finally, CERS3 synthesizes C28-32 ceramides which are exclusively expressed in skin and testes (19). C16 in HNSCC cells has been shown to act in a protective manner, preventing these cells from ER stress-mediated apoptosis (20), while C16 has also been shown to be responsible for radiation-mediated apoptosis in HeLa cells.

These findings suggest that the tissue specificity plays a role in not only the expression of the various ceramide-generating enzymes and the subsequent chain length of the generated ceramides, but also that the function in cellular proliferation is a tissue specific phenomenon. Therefore, when targeting these enzymes with chemotherapeutics, one must consider the cellular specificity of the drug, the target, and the acyl length of the ceramide species in question.

### Sphingolipid Involvement in Cellular Fate

Apoptosis may be initiated in a cell via one of two pathways. The first pathway, known as both the intrinsic pathway and the mitochondrial death pathway, utilizes the B-cell lymphoma-2 (Bcl-2) regulator proteins located on the outer mitochondrial membrane to signal for a cell to undergo apoptosis once a proper stimulus is detected. This signal cascade ultimately results in an increased permeability of the mitochondrial membrane, allowing a cascade of the caspase proteins, ending with caspase 3 and caspase 7 destroying the cell from within. The second pathway, known as both the extrinsic pathway and the death receptor pathway, utilizes a TNF death receptor protein found on the surface of cells that is encoded by the FAS gene. The extrinsic pathway also causes a caspase cascade that ultimately results in apoptosis via caspase 3.

Ceramide is a critical mediator of both intrinsic and extrinsic mechanisms of apoptosis. Ceramide's role in the extrinsic pathway is achieved by mimicking the cytotoxicity of TNF (21). Ceramide's many roles in apoptosis are a heavily studied and controversial area of research, but the best described proapoptotic mechanism involves the intrinsic pathway of cell death. Intrinsic apoptotic stimuli have been show to modify enzymes involved in the synthesis and degradation of ceramide, ultimately leading to increased ceramide levels, which can then go on to function as a second messenger in the intrinsic pathway downstream of Bcl2 via a variety of actions (22). Ceramide aggregates can also form channels on the outer mitochondrial membrane independent of the BCL2 proteins, directly leading to OMM permeability, signal cascade, and ultimately cell death (23). S1P directly opposes the proapoptotic effects of ceramide by binding to S1PR, which stimulates downstream cleavage of caspase-3, halting the signal cascade (24).

## Agents Targeting Sphingolipid Pathways

### Inhibitors of Ceramide Degradation

#### FTY720

FTY720 (Fingolimod) is a sphingosine analogue that is an FDA approved immunosuppressant for the treatment of MS but has since been shown to have some efficacy as an antitumor medication (25). Unlike its use as an immunosuppressant, when used as an anticancer medication, FTY720 does not require the phosphorylation via SphK2 (26). Instead, FTY720 utilizes a myriad of mechanisms to ultimately promote cell death such as inhibition of SphK1 and S1PR internalization, leading to complex downstream effects.

While Fingolimod has shown potential both *in vivo* and *in vitro* for treating various malignancies, the immunosuppressive properties it possesses likely restrict its practical applications in the clinic and thus its entrance into clinical trials as a monotherapy for cancer. However, there are several potential methods proposed to overcome this hurdle. One such method is to prevent the phosphorylation of this drug via the development of a synthetic derivative which may not be phosphorylated and therefor may not display any immunosuppressive properties. OSU-2S (27) is a derivative of FTY720 that is immune to phosphorylation via SphK2, thus this drug warrants further research. Another method is to carefully control tissue specific delivery of FTY720 via the development of liposomal carriers such that the drug cannot have its phosphorylated form enter the peripheral circulation (28). Alternatively, the immunosuppressive functions of FTY720 may be exploited in certain patient populations such as patients with posttransplant malignancies in which immunosuppression would be a favorable complement to anticancer therapy. Additionally, studies are investigating Fingolimod with radiation and temozolomide in treatment of high-grade glioma to reduce radiation-related lymphopenia. In this application FTY720 functions to induce lymphopenia prior to the beginning of radiation treatment such that inadvertent radiation of circulating lymphocytes is reduced, allowing a reversible lymphopenia after discontinuing FTY720 (NCT02490930). This study has recently completed Phase I clinical trials. Table 1 summarizes the agents targeting sphingolipid pathway that are currently under development in preclinical or clinical studies (27, 29–64).

**Table 1:** Antineoplastic agents targeting sphingolipids that are in development.

**Agents in Clinical Trials**					
**Antineoplastic**	**Target**	**Developing Company**	**Clinical Trial Status**	**Cancer treated**	**References**
FTY720 (Fingolimod)	S1PR, SphK1	Novartis	Phase 1a completed, not recommended to progress	Glioma	NCT02490930
Sonepcizumab (Asonep)	S1P	Lpath Therapeutics, Inc.	Phase II completed	Refractory renal cell carcinoma	(29)
			Phase II terminated	advanced solid tumors	NCT01762033
ABC294640 (Yeliva)	SphK2, DES	RedHill Biopharma	Phase II in progress	Cholangiocarcinoma	NCT03377179
			Phase II in progress	Adv HCC	NCT02939807
			Phase I completed	Adv Solid Tumors	(30)
			Phase I withdrawn	Diffuse large B-cell Lymphoma	NCT02229981
			Phase I terminated	Multiple Myeloma	NCT02757326
fenretinide	targets being investigated^**^	CerRx, Inc.	Phase II in progress	Relapsed peripheral T-cell lymphoma^*^	NCT02495415^*^
Desipramine (Norpramin)	Acid Ceramidase	Novartis	Phase IIa terminated	SCLC/high-grade neuroendocrine tumors	NCT01719861
			n/a	Multiple myeloma	NCT01899326
Nanoliposome ceramides	n/a	Keystone Nano, Inc	Phase I in progress	Advanced solid tumors	NCT02834611
Safingol (Kynacyte)	PKC, SphK1	South Plains Oncology Consortium	Phase I in progress	Relapse malignancies	NCT01553071
				Metastatic solid tumors	NCT00084812
KW2871 (Ecromeximab)	GD3	Kyowa Hakko Kogyo Co., Ltd	Phase I complete	Metastatic melanoma	(31)
			Phase II terminated	Advanced metastatic melanoma	NCT00199342
Fluphenazine	ASMase		Phase II completed, no results posted	Multiple Myeloma	NCT00335647
**Antineoplastic**	**Target**	**Developing Company**	**Clinical Trial Status**	**Cancer models**	***in vitro*****/*****in vivo***	**References**
**Agents in Preclinical Studies**						
*B13*	Acid Ceramidase	n/a	preclinical	Melanoma, pancreatic cancer, metastatic colon cancer, prostate	*in vitro*	(32)
*LCL204*	Acid Ceramidase	n/a	preclinical	Prostate	*in vitro*	(33)
*LCL385*	Acid Ceramidase	n/a	preclinical	Prostate	*in vitro*	(34)
*AD2646*	Acid ceramidase	n/a	preclinical	Prostate, melanoma	*in vitro*	(35)
*AD2687*	Ceramide analogue	n/a	preclinical	Leukemia	*in vitro*	(36)
*LCL464*	Acid ceramidase	n/a	preclinical	Breast	*in vitro*	(37)
*n-oleoylethanolamine*	Acid ceramidase	n/a	preclinical	Hepatoma	*in vivo*	(38)
*D-MAPP*	Acid ceramidase Neutral ceramidase	n/a	preclinical	Squamous cell carcinoma	*in vitro*	(39)
*Dimethylsphingosine*	Sphingosine kinase	n/a	preclinical	Leukemia, pheochromocytoma	*in vitro*	(40)
*Trimethylsphingosine*	PKC	n/a	preclinical	Melanoma	*in vitro*	(41)
*Dihydroxyaurone*	SphK1	n/a	preclinical	Breast	*in vitro*	(42)
*BML-258 (SK1-I)*	SphK1	n/a	preclinical	Leukemia	*in vivo*	(43)
*D609*	Sphingomyelin synthase	n/a	preclinical	Leukemia	*in vitro*	(44, 45)
*C18 Pyridinium*	Exogenous ceramide	n/a	preclinical	HNSCC	*in vitro*	(46)
*PDMP*	Glucosylceramide synthase	n/a	preclinical		*in vitro*	(47)
*C16-serinol*	Exogenous ceramide	n/a	preclinical		*in vitro*	(48)
4,6-ketone-diene-ceramide	Exogenous ceramide	n/a	preclinical	Breast, ovarian	*in vitro*	(49)
5R-OH-3E-C8-ceramide	Exogenous ceramide	n/a	preclinical	Breast	*in vitro*	(50)
Adamantly-ceramide	Exogenous ceramide	n/a	preclinical	Breast	*in vitro*	(50)
Benzene-c4-ceramide	Exogenous ceramide	n/a	preclinical	Breast	*in vitro*	(50)
C6-pyridinium-ceramide (LCL29)	Exogenous ceramide	n/a	preclinical	HNSCC insulinoma	*in vivo*	(46, 51)
LCL-30	Exogenous ceramide	n/a	preclinical	Colorectal	*in vivo*	(52)
JTE-013	S1PR	n/a	preclinical	Glioma	*in vivo*	(53)
AB1	S1PR2	n/a	preclinical	Glioma glioblastoma	*in vivo*	(53)
PF543	SphK1	n/a	preclinical	Colorectal	*in vivo*	(54)
VPC03090	S1PR	n/a	preclinical	Breast	*in vivo*	(55)
F-12509A	SphK1	n/a	preclinical	Acute myeloid leukemia	*in vitro*	(56)
LCL146	SphK1	n/a	preclinical	Colon	*in vitro*	(57)
LCL351	SphK1	n/a	preclinical	Colon	*in vitro*	(57)
SKI-178	SphK	n/a	preclinical	Acute myeloid leukemia	*in vivo*	(58)
PF-543	SphK1	n/a	preclinical	Colorectal, gastric	*in vitro*	(59)
C8-cyclopropenylceramide	Dihydroceramide desaturase	n/a	preclinical	neuroblastoma	*in vitro*	(60)
NVP-231	CerK	n/a	preclinical	Breast, lung	*in vitro*	(61)
LCL521	Acid Ceramidase	n/a	preclinical	Breast adenocarcinoma	*in vitro*	(62)
LCL204	Acid ceramidase	n/a	preclinical	Prostate	*in vivo*	(33)
GW4869	Neutral sphingomyelinase 2	n/a	preclinical	Breast	*in vitro*	(63, 64)
OSU-2S	S1PR	n/a	preclinical	HCC	*in vitro*	(27)

* *Multiple clinical trials ongoing, found at https://www.drugbank.ca/drugs/DB05076*.

** *Summary of fenretinide mechanism of action https://www.ncbi.nlm.nih.gov/pubmed/25069047*.

#### ABC294640

ABC294640 is a competitive inhibitor of SphK2 thus it alters the rheostat in favor of pro-apoptotic ceramide, and depletion of pro-survival S1P thereby promoting both autophagy as well as apoptosis (65). Unexpectedly, cells that are treated with ABC294640 show increased dihydroceramides, suggesting that this drug also inhibits dihydroceramide desaturase (66). In preclinical studies, ABC294640 demonstrated excellent activity in xenograft models of hepatocellular carcinoma and reduced plasma S1P by up to 50% at tolerable doses (67). These studies also concluded a satisfactory oral bioavailability, and found few adverse reactions associated with even the highest doses administered. Phase I clinical trials expanded further on the safety of this drug and found adverse effects to be mild at therapeutic doses, although some patients experienced neuropsychiatric symptoms that were reversible with discontinuation of the therapy (30).

ABC294640 is a novel therapeutic that is among the first drug of any kind to target sphingolipid metabolism. Phase I trials demonstrated tolerability of the drug, established dosing, and proved efficacy of antitumor properties. Preclinical studies have shown its potential in treating lung, prostate, liver, ovarian and colorectal cancers thus we can expect many clinical trials to further explore ABC294640. Phase II studies are currently ongoing for using ABC294640 to treat large B-cell lymphoma (NCT02229981), multiple myeloma (NCT02757326), cholangiocarcinoma (NCT03414489), as well as hepatocellular carcinoma (NCT02939807), with each study in various stages of completion.

#### 4HPR (Fenretinide)

Fenretinide is a synthetic retinoid that has been shown to possess apoptotic effects in a variety of malignant cells including leukemia, neuroblastoma, lung cancer, cervical carcinoma, bladder cancer, HNSCC, and prostate cancer cells (68). While the mechanism of action is yet to be fully understood, several targets have been identified. Of the most relevant mechanisms proposed, in neuroblastoma, cell death independent of caspase cascade was observed via an increase in *de novo* ceramide synthesis (69). However, many other proposed mechanisms exist including generation of reactive oxygen species (ROS) (70), activation of c-Jun N-terminal kinase signaling, and inhibition of expression of COX-2 causing diminished prostaglandin synthesis (71).

Phase I clinical trials studying fenretinide were carried out in 31 adults and 50 children. The group of children presented with adverse effects such as increased intracranial pressure, hypoalbuminemia, hypophosphatemia, and elevated transaminases. In adults, adverse effects include dry skin, nyctalopia, hepatic dysfunction, among some less severe symptoms such as nausea and vomiting. While phase II clinical trials failed to prove the efficacy of fenretinide in bladder cancer (72) low bioavailability has been limiting its therapeutic potential, which may be addressed via inhibition of oxidizing liver enzymes such as CYP3A4 with drugs such as ketoconazole (73).

#### Desipramine

Desipramine is an FDA approved tricyclic antidepressant that is used to treat a decrease in the bioavailability of monoamines by inhibiting synaptic reuptake, with evidence also pointing to a decrease in the production of neuronal TNF-α (74). In 2006, studies provided evidence that desipramine also has antitumor properties in multiple cancer cell lines via a dose-dependent downregulation of acid ceramidase, increasing ceramide levels, and inducing apoptosis (75). With prior FDA approval and preclinical evidence of antitumor activity, primarily in the exceedingly fatal disease small-cell lung cancer, researchers were eager to proceed with clinical trials to quickly move this drug from the laboratory to the clinic. Despite demonstrated tolerability of the drug and promising preclinical efficacy, phase IIa clinical trials were terminated when desipramine failed to prove efficacious in treating small-cell lung cancer and other neuroendocrine tumors (NCT01719861).

#### Safingol

Sphingosine and lysosphingolipids are potent inhibitors of protein kinase C (PKC), a component of numerous signal transduction pathways that promote cell activation and tumorigenesis (76). Safingol is a saturated derivative of sphingosine that competitively inhibits PKC via binding at the phorbol-binding domain (77). Additionally, Safingol may have SphK inhibition properties as well, promoting the accumulation of ceramide and inhibiting the generation of S1P; cumulatively these effects lead to a caspase independent cell death via autophagy due to downstream disruption of PI3k/mTOR pathway and the MAPk pathway (77).

Multiple clinical trials have been completed evaluating the safety profile of Safingol, each of which included an accompanying antineoplastic. A phase I clinical trial of Safingol with Cisplatin for treating advanced solid tumors found both the drug combination as well as Safingol monotherapy to be relatively safe, with reversible, dose-dependent hepatotoxicity being the most severe adverse effect observed (78). An ongoing Phase I clinical trial is being conducted to determine the safety of combining Safingol with Fenretinide (NCT01553071), a promising drug combination due to the synergistic effects shown in preclinical studies, owing to Safingol's ROS generating properties (79).

### Agonists of Ceramide Synthesis

#### Fluphenazine

Fluphenazine is a phenothiazine antipsychotic approved by the FDA to treat schizophrenia and psychotic symptoms such as delusions and hallucinations. As an antipsychotic, the mechanism of action of Fluphenazine is thought to be via an interruption of dopamine neurotransmission in the brain, but off target effects include inhibition of acid sphingomyelinase causing an accumulation of sphingomyelin (80). The accumulation of sphingomyelin is particularly pronounced in hypoxic tumors, a common site of resistance to antiproliferative chemotherapeutics (81), thus Fluphenazine may be useful in treating solid tumors that are typically resistant to chemotherapy. Interestingly, multiple myeloma tumor progression induces hypoxic conditions in bone marrow, activating proteins involved in the epithelial-mesenchymal transition and ultimately promoting metastasis of multiple myeloma cells (82). Accordingly, clinical trials involving fluphenazine investigated this drug's usefulness in treating multiple myeloma (NCT00821301).

### Molecular Mimics/Endogenous Sphingolipids

#### Nanoliposomal Ceramide

Introducing exogenous ceramides into cells has long been proposed as a method to induce apoptosis in cancerous cells. However, being a long acyl chain containing molecule, endogenous ceramides pose significant issues in the route of administration, owing to their inherent hydrophobicity and insolubility. This first limitation was recognized in a phase II clinical trial utilizing a topical C6 ceramide cream to treat breast cancer; a lack of efficacy shelved the topical use of ceramides but verified their lack of toxicity (NCT00008320). Nanoliposome ceramides overcome this hurdle by utilizing a short chain ceramide, C6, carried in pegylated nanoliposomes (83). First shown to be effective in slowing the growth of hepatic tumors in mice (84), nanoliposomal ceramide has since begun a currently ongoing phase I clinical trial for patients with advanced solid tumors (NCT02834611).

### Sphingolipids as Targets

#### Sonepcizumab

Sonepcizumab is an anti-S1P Ab drug therapy that has completed phase I clinical trials (NCT00661414) and has recently had terminated Phase II (NCT01762033) clinical trials for treating refractory renal cell carcinoma. High specificity has been demonstrated for S1P, even over other bioactive lipids such as sphingosine. This high specificity is due to its preferential recognition of a phosphorylated amino-alcohol carried via the head of a sphingosine base, thus not even other substitutions for the phosphate such as D-galactose will trigger binding of sonepcizumab. As one would expect, the neutralization of S1P has been shown to decrease cancer progression via tumor angiogenesis inhibition, starving the cancerous cells of nutrients necessary for proliferation. For example, in mice models sonepcizumab inhibited xenograft angiogenesis even though mice have plasma S1P significantly higher than that of humans, suggesting that even when antigen is abundant, therapy with this drug still was able to inhibit angiogenesis in tolerable doses (85).

Targeting dysregulated S1P is a common goal of many of the chemotherapeutics discussed in this review, but few have shown to work with such specificity, efficacy, and safety as does sonepcizumab's early clinical trials suggest. Despite sonepcizumab's failure to meet progression-free survival endpoints in treating RCC, the safety and overall survival rate demonstrated in this trial suggests the need for future studies of this drug in combination with other antineoplastic medications. As future studies proceed to completion, it is likely that this drug will be among the first monoclonal antibodies against a bioactive lipid to be utilized in the clinic.

### Ecromeximab (KW2871)

KW2871 is a monoclonal antibody targeting the GD3 ganglioside, a prominent cell surface antigen on malignant melanoma cells (86). Clinical trials combining KW2871 with interferon-α2b, a protein antineoplastic, were recently completed in 2018. Despite phase I trials demonstrating an acceptable safety profile with few adverse events, phase II trials of this drug combination failed to prove the efficacy in treating metastatic melanoma (NCT00679289). However, other combinations with this drug will likely be tested in clinical trials, as KW2871 showed excellent activity in preclinical studies by extrapolated studies from the mouse GD3 monoclonal antibody R24 (87), and better drugs such as immune checkpoint inhibitors (88) are being developed and are thought to have potential synergism with KW2871.

## Existing Anticancer Drugs Found To Involve Sphingolipids

Numerous existing anticancer agents have been shown to induce increased levels of endogenous ceramides, which may be involved in their mechanism of promoting cancer cell death. Cytarabine, a drug used to treat acute myeloid leukemia, has been shown to increase ceramide levels via *de novo* synthesis (89). The classic chemotherapy drugs anthracyclines, etoposide and paclitaxel have each been shown to induce acid sphingomyelinase (90), leading to increased ceramide from the hydrolysis of sphingomyelin. However, it is unclear whether or not the activation of sphingomyelinase in each of these instances is responsible for inducing apoptosis, but rather may just be a response to cellular stress (91).

Tamoxifen, a well-known anticancer agent for Her2 breast cancer, has been an attractive agent as an adjuvant for ceramide-based therapies, for it has been identified as a glucosylceramide synthase inhibitor (92). P-glycoprotein is a drug efflux transporter found to be overexpressed in numerous multidrug resistant cancers, particularly to ceramide-based therapies and to the drugs paclitaxel, vinblastine, vincristine, daunorubicin, doxorubicin, and etoposide (93). In a complex interplay between ceramide glycosylation, ceramide induced apoptosis, and p-glycoprotein, studies have shown that expression of p-glycoprotein confers resistance to ceramide toxicity (94), and that overexpression of glucosylceramide synthase and P-glycoprotein in cancer cells selected for resistance chemotherapy (95). These findings imply that GCS inhibitors such as Tamoxifen, vincristine, doxorubicin, and Taxol will likely function as a sensitizer to drug resistant cancers (90). Indeed, this topic has been studied extensively with many completed and ongoing clinical trials in various stages of progression.

## Conclusions

In healthy cells, sphingosine and ceramide play a significant role in cellular apoptotic machinery while S1P accumulation leads to cell proliferation, angiogenesis, and mediates the inflammatory response. But in cancerous cells, both over and under expression of enzymes involved in the metabolism of sphingolipids induces aberrant expression of sphingosine kinases, ceramide degrading enzymes, or S1PRs ultimately altering cellular fate to favor mitogenic, pro-survival states. These pathways are believed to be potentially important therapeutic targets in the goal of treating cancer, but with new targets come new risks and new questions that need to be answered. Without a comprehensive understanding of the metabolic processes, feedback regulation, and downstream targets of sphingolipid metabolism, targeting sphingolipid metabolism is a still only a conjecture in the ultimate goal, which may nonetheless prove to be worthwhile.

## Author Contributions

AK conducted a literature search, wrote the draft of the mini-review. HC and MK reviewed and revised the manuscript.

## Conflict of Interest

The authors declare that the research was conducted in the absence of any commercial or financial relationships that could be construed as a potential conflict of interest.
